# The Prevention of Cholera

**Published:** 1919-05-10

**Authors:** 


### The Prevention of Cholera.

The history of great wars in the past illustrates the frequency with which war conditions are associated with, or followed by, serious outbreaks of epidemic disease. At the present time cholera is reported in Berlin and Vienna and is present to some considerable extent in Russia and the adjoining countries and while it is extremely unlikely that any serious outbreak will occur in this country, it is not improbable that isolated cases of this disease may crop up and provide a possible source of infection. It is necessary, therefore, that Public Health Authorities, more especially at ports of possible entry, should exercise increasing vigilance with regard to the presence of any suspected case so that immediate and adequate precautionary measures may be taken. Cholera is endemic in India, and on several occasions during last century it appeared in epidemic form in Europe and invaded England. The last occasion on which cholera appeared in England in epidemic form was in 1865-6, and although on subsequent occasions it again invaded Europe, England escaped except for a few isolated cases. Moreover, since the last serious invasion of cholera enormous strides have been made in sanitation and in the providing of pure and protected water supplies. Cholera is essentially a disease which is spread by contaminated drinking-water; but in populous districts, where there are over-crowding and insanitary home conditions, infection may be spread by personal contact, infected material, and flies. Owing to the enlightened action of health authorities with regard to water supplies there is now little likelihood of any serious outbreak resulting from contaminated drinking-water, while the risk of local outbreaks from any isolated cases which might occur can be greatly minimised by maintaining a high hygienic standard in the home.

